# Pediatric low-grade glioma in the era of molecular diagnostics

**DOI:** 10.1186/s40478-020-00902-z

**Published:** 2020-03-12

**Authors:** Scott Ryall, Uri Tabori, Cynthia Hawkins

**Affiliations:** 1 Arthur and Sonia Labatt Brain Tumour Research Centre, The Hospital for Sick Children, 555 University Avenue, Toronto, ON M5G 1X8 Canada; 2 Department of Laboratory Medicine and Pathobiology, University of Toronto, Toronto, ON Canada; 3 Division of Pathology, The Hospital for Sick Children, Toronto, ON Canada; 4 Division of Haematology and Oncology, The Hospital for Sick Children, Toronto, ON Canada; 5 Department of Medical Biophysics, University of Toronto, Toronto, ON Canada

**Keywords:** Pediatric low-grade glioma, glioma, pediatric, neurofibromatosis type I, brain tumor, neuro-oncology, RAS/MAPK pathway, molecular diagnostics, clinical trial, targeted therapy, risk stratification

## Abstract

Low grade gliomas are the most frequent brain tumors in children and encompass a spectrum of histologic entities which are currently assigned World Health Organisation grades I and II. They differ substantially from their adult counterparts in both their underlying genetic alterations and in the infrequency with which they transform to higher grade tumors. Nonetheless, children with low grade glioma are a therapeutic challenge due to the heterogeneity in their clinical behavior – in particular, those with incomplete surgical resection often suffer repeat progressions with resultant morbidity and, in some cases, mortality. The identification of up-regulation of the RAS–mitogen-activated protein kinase (RAS/MAPK) pathway as a near universal feature of these tumors has led to the development of targeted therapeutics aimed at improving responses while mitigating patient morbidity. Here, we review how molecular information can help to further define the entities which fall under the umbrella of pediatric-type low-grade glioma. In doing so we discuss the specific molecular drivers of pediatric low grade glioma and how to effectively test for them, review the newest therapeutic agents and their utility in treating this disease, and propose a risk-based stratification system that considers both clinical and molecular parameters to aid clinicians in making treatment decisions.

## Introduction

**Fig. 1.**
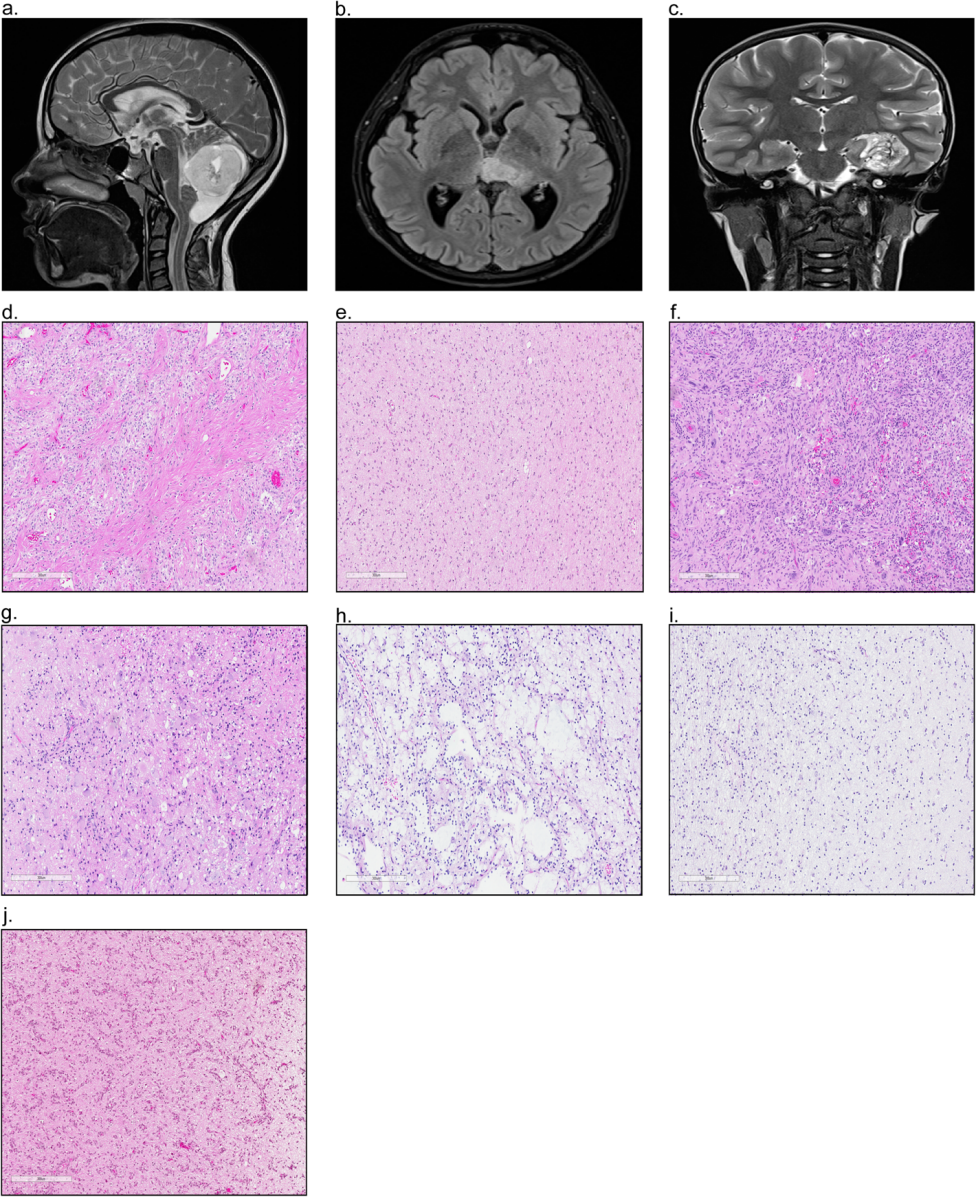
Magnetic resonance imaging (MRI) depicting pediatric low-grade glioma arising in the **a**. Cerebellum, **b**. Thalamus, and **c**. Occipital Lobe. Hematoxylin and eosin (H&E) staining highlighting the hallmark histologic features of **d**. Pilocytic astrocytoma, **e**. Diffuse astrocytoma, **f**. Pleomorphic xanthoastrocytoma, **g**. Ganglioglioma, **h**. Dysembryoplastic neuroepithelial tumour, **i**. Oligodendroglioma, and **j**. Angiocentric glioma

Tumors of the central nervous system (CNS) are the most frequent solid tumors in children, with approximately 5.4-5.6 diagnoses per 100,000 [48, 154, 155]. Of those diagnosed, 0.7 per 100,000 will succumb to their disease, making CNS tumors the leading cause of cancer related death in children [154, 155, 168]. Within this group, pediatric-type low-grade gliomas (pLGG) are the most frequent, accounting for approximately 30% of all childhood brain tumors [154, 155]. pLGG are defined as World Health Organization (WHO) grade I or II malignancies and encompass a wide array of histologies that can arise throughout the neuro-axis **(**Fig. 1a-c) [131, 132].

Management of pLGG is intimately related to surgical resection, and complete resection remains the most favorable predictor of patient outcome [225]. Often, this is achievable for superficial lesions such as those arising in the cerebral hemispheres or posterior fossa, but is not always feasible for deep seated or highly infiltrative tumors [225]. In these cases, progressive residual disease has historically been treated with adjuvant chemotherapy or radiation [12, 55, 122, 124, 135, 139, 157, 183, 189]. Importantly, these treatments are associated with long-term sequelae and, particularly for radiation, increased mortality [55, 71, 118, 138, 145]. These concerns are poignant in a disease where 10-year overall survival (OS) exceeds 90%. However, with progression-free survival (PFS) at approximately 50%, up to half of patients will require adjuvant therapy. As such, a more robust risk stratification is required to help guide the type and intensity of therapy warranted. In the past, the degree of surgical resection, histological diagnosis and age were used to determine prognosis. However, more recently the molecular underpinnings of pLGG have emerged as a powerful tool to supplement the stratification of these tumors.

In the last decade, significant molecular data has emerged to suggest that pLGG near universally up-regulate the RAS-mitogen-activated protein kinase (RAS/MAPK) pathway [34, 96, 149, 229]. This data has led to increasing use of targeted therapeutics that supplement and/or replace older cytotoxic approaches. As the era of targeted therapeutics inevitably arrives, a concise classification scheme recognizing the molecular features of pLGG is needed. Here, we will review the histological spectrum of pLGG, the molecular alterations that have been identified in these entities and how to effectively test for them, and review the newest therapeutic agents and their utility in treating this disease. We conclude with proposing a multi-faceted approach for stratifying pLGG that considers clinical, histologic and molecular parameters and aims to aid clinicians in their future treatment decisions.

## Morphologic Classification of pLGG

pLGG form a heterogeneous group of neoplasms that encompass tumors of primarily glial histology, including astrocytic and/or oligodendroglial, and tumors of mixed neuronal-glial morphology. These tumors are considered grades I and II according to the current WHO classification where they are distinguished from high grade glioma on the basis of specific morphologic features or, in the case of diffuse glioma, based on the absence of necrosis, mitoses and microvascular proliferation [131, 132, 166, 199]. Histologic diagnoses which fall under the umbrella of pLGG and their hallmark molecular alterations are listed in Table 1; their typical histologic features are depicted in Fig. 1d-j.

**Table 1:** Histological diagnosis and the common molecular events of WHO-recognized pLGG. RTK: receptor tyrosine kinase, SNV: single nucelotide variant

Histological Diagnosis	Common Molecular Events
Glial Tumors	
Pilocytic Astrocytoma	*KIAA1549-BRAF* (70-80%) *FGFR1-TACC1* (3-5%) *FGFR1* SNV (3-5%) BRAF p.V600E (3-5%) Other *BRAF* Fusions (2-5%) *CRAF* Fusions (2-5%) *PTPN11* SNV (2-5%) *KRAS/HRAS* SNV (2-5%)
Subependymal Giant Cell Astrocytoma	*TSC1/2* SNV (85-95%)
Diffuse Astrocytoma	BRAF p.V600E (20-40%) *MYBL1* alteration (5-10%) *KIAA1549-BRAF* (5-10%) *FGFR1* SNV (2-5%) H3.3 p.K27M (2-5%) IDH1 p.R132H (2-5%) Other RTK SNV/Fusions (2-3%)
Pleomorphic Xanthoastrocytoma	BRAF p.V600E (80-90%)
Oligodendroglioma	*FGFR1-*TKD duplication (10-20%) *FGFR1* SNV (10-20%) BRAF p.V600E (5-10%) *FGFR1-TACC1* (3-5%) IDH1 p.R132H (3-5%) 1p/19q co-deletion (3-5%)
Mixed Glioneuronal Tumors	
Ganglioglioma	BRAF p.V600E (40-50%) *KIAA1549-BRAF* (10-15%)
Desmoplastic Infantile Astrocytoma and Ganglioglioma	BRAF pV600E/D (40-60%) *FGFR1* SNV (5-10%) *KIAA1549-BRAF* (2-5%)
Dysembryoplastic Neuroepithelial Tumor	*FGFR1-*TKD duplication (20-30%) *FGFR1* SNV (20-30%) *FGFR1-TACC1* (10-15%) Other RTK SNV/Fusions (5-10%) BRAF p.V600E (5-10)
Papillary Glioneuronal Tumor	*SLC44A1-PRKCA* (80-90%)
Rosette-forming Glioneuronal Tumor	*PIK3CA* SNV (20-30%) *KIAA1549-BRAF* (20-30%) *FGFR1* SNV (20-30%)
Angiocentric Glioma	*MYB* (80-90%)
Chordoid Glioma of Third Ventricle	*PRKCA* SNV (80-90%)
Polymorphous Low-Grade Neuroepithelial tumor of the Young (PLNTY)	BRAF p.V600E (30-40%) *FGFR2/3* Fusions (30-40%)
Multinodular and vacuolating neuronal tumor (MVNT)	*MAP2K1* SNV/Indel (50-60%) BRAF p.V600E (5-10%) Other *BRAF* SNV (5-10%) *FGFR2* Fusions (3-5%)

In many cases the different histologic entities are readily distinguished, however cases of overlapping morphology are well documented. These include, for example, reports of histological overlap between pleomorphic xanthoastrocytoma and ganglioglioma [3, 62, 208] and between dysembryoplastic neuroepithelial tumor and oligodendroglioma [70, 114]. In addition, tumors which are classically well circumscribed, such as pilocytic astrocytoma, may possess an infiltrative component [34], leading to confusion and difficulty in grading. A precise histologic diagnosis may be particularly challenging in deep seated midline tumors, for which a small biopsy is often all that is available. Rarely do these capture the true complexity of the tumor and the classic morphologic features by which diagnoses are made are often lacking.

In addition to these difficulties, pLGG overlap morphologically with entities more commonly found in adults. This creates confusion regarding appropriate grading and treatment and is exacerbated by use of similar terminology, namely diffuse astrocytoma and oligodendroglioma. In the most recent WHO iteration, both diffuse astrocytoma and oligodendroglioma have been split based on the presence or absence of *IDH1* mutations, in addition to 1p/19q co-deletion for the latter. Tumors with the morphology of oligodendroglioma or diffuse astrocytoma in the pediatric age group often do not have *IDH1* mutations and/or 1p/19q co-deletion and are therefore considered oligodendroglioma, NEC or, of even greater concern, diffuse astrocytoma, *IDH*-wildtype. The latter raising concern for molecular glioblastoma (GBM). Both of these diagnoses may lead to conventional adult diffuse glioma treatments involving cytotoxic chemotherapy and radiation, particularly in the adolescent and young adult age group. However, in *IDH1* wild-type cases, pediatric oligodendrogliomas most frequently harbor alterations in *FGFR1* including TKD-duplications or SNVs or BRAF p.V600E (Table 1). Recently, the entity polymorphous low-grade neuroepithelial tumor of the young (PLNTY) was described [88]. These tumors invariably possessed oligodendroglioma-like cellular components and highly infiltrative morphological features, yet boast a benign clinical course uncommonly seen in classic *IDH*–mutant oligodendroglioma [31, 88]. These tumors do not harbor *IDH1* mutations, but rather *FGFR2/3* fusions (discussed further below) or BRAF p.V600E. *IDH1* wild-type diffuse astrocytoma most frequently harbor BRAF p.V600E mutations, accounting for ~40% of cases (Table 1). In addition, they sometimes contain *KIAA1549-BRAF* fusions, *FGFR1* SNVs, or *MYB* or *MYBL1* alterations. The latter alterations were originally described in series of pediatric diffuse astrocytomas [174, 212] and reports thus far suggest they have a benign clinical course without therapy [31]. Recently, these have been termed isomorphic diffuse glioma [223]. In these cases, misdiagnosis may result in over-treatment, leading to potentially harmful sequelae.

In recognition of the increased understanding of the molecular underpinnings of diffuse gliomas in adults, *IDH1* mutation and 1p/19q deletion status were incorporated into the most recent WHO revision in order to improve diagnostic reproducibility and provide important prognostic information for patients [132]. A similar incorporation of molecular features into the classification of pLGG will help to more accurately identify these entities and, importantly, distinguish them from adult-type gliomas, which carry a worse prognosis and require more aggressive therapy.

## The Molecular Landscape of pLGG

### Up-regulation of the RAS/MAPK Pathway

**Fig. 2.**
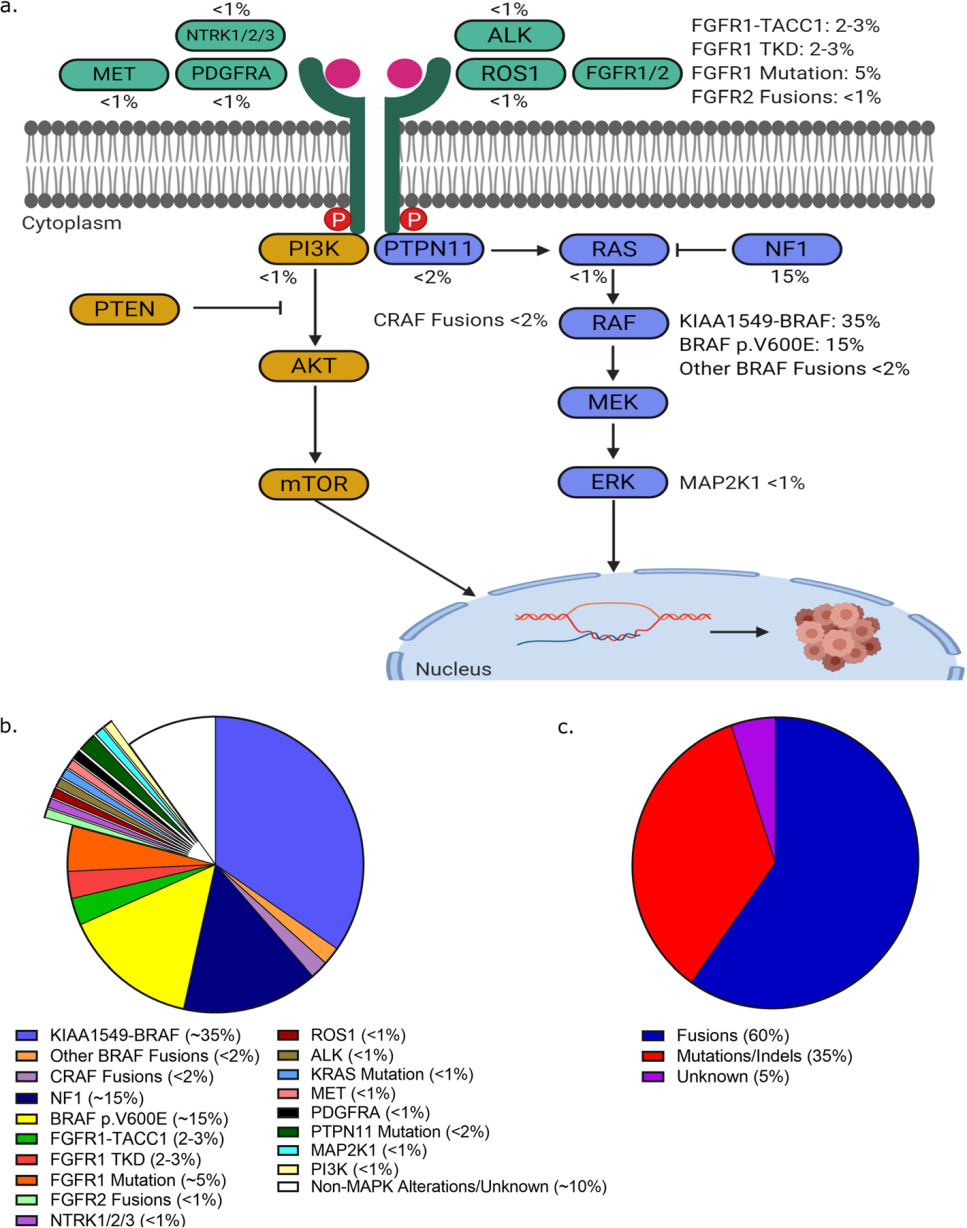
**a**. Schematic of the RAS/MAPK alterations identified across pediatric low-grade glioma. **b**. Average frequencies of RAS/MAPK alterations identified in pediatric low-grade glioma at the population level. **c**. Alteration types identified in pediatric low-grade glioma

The last decade has produced unparalleled insights into the underlying biology of pLGG. Importantly, we now know that the majority of pLGG are driven by a single genetic event resulting in up-regulation of the RAS/MAPK pathway [34, 96, 149, 229]. Our first indications of RAS/MAPK involvement in pLGG pathogenesis came from Neurofibromatosis Type I (NF1) patients of whom 10-15% develop low-grade glioma [14, 196, 218]. Since then, molecular profiling efforts have uncovered additional alterations within this pathway with such frequency that many have postulated that pLGG is a "one-pathway disease" [34, 96, 149, 229]. An overview of the most common RAS/MAPK pathway alterations in pLGG is shown in Fig. 2.

### Neurofibromatosis Type 1

Neurofibromatosis Type I (NF1) is the most common inheritable tumor predisposition syndrome worldwide and is associated with a wide range of clinical manifestations including skin pigmentation abnormalities, learning disabilities, seizures, and vasculopathies [16, 19]. NF1 is caused by a germline mutation in the *NF1* tumor suppressor gene, which encodes neurofibromin, a GTPase-activating protein that functions as a negative regulator RAS [61, 89, 177]. 10-15% of children with NF1 will develop a low-grade glioma within the optic pathway, with an additional 3-5% arising outside of the optic pathway [14, 195, 196, 218]. NF1-associated gliomas usually show loss of the wild-type allele and, as a result, neurofibromin's endogenous function as a negative regulator of RAS is lost. Typically, NF1-pLGG are asymptomatic and indolent, requiring no therapeutic intervention and in some cases, regress without treatment [87, 119, 126, 127, 159]. However, in cases of clinical deterioration (most commonly vision loss), chemotherapy, and not radiation, is the first line of treatment [87, 119, 126, 127, 159, 197].

Despite their benign course, NF1-pLGG arising in younger children (<2 years) and/or outside of the optic pathway are recognized as being at a higher risk of progression and/or death [59]. Historically, NF1-pLGG are not biopsied due to their precarious location and the lack of clinical utility of the additional information obtained. However, a recent study uncovered that NF1-pLGG do harbor additional genetic alterations [37]. Most commonly, these were additional aberrations affecting the RAS/MAPK pathway or those involving transcriptional regulators. Furthermore, the mutational profile of NF1-pLGG was distinct from NF1-high grade glioma (HGG), which instead harbored alterations in *TP53*, *CDKN2A* and *ATRX*. Therefore, obtaining a biopsy from, at minimum, patients deemed higher risk, may prove valuable in identifying patients that require refined and/or novel treatments and distinguishing them from NF1-HGG, particularly in adults.

#### KIAA1549-BRAF

Early studies examining copy number alterations in pilocytic astrocytoma identified focal gains at 7q34 which included the *BRAF* gene [44, 167]. Further work by Jones *et. al.* refined this discovery, showing that this gain was the result of a tandem duplication resulting in the formation of a novel oncogenic fusion, *KIAA1549-BRAF* [99]. This rearrangement resulted in the N-terminal regulatory domain of *BRAF* being lost, leading to downstream up-regulation of the RAS/MAPK signaling pathway [99]. Five separate *KIAA1549-BRAF* exon-exon junctions have been described including 16;9, 15;9, 16;11, 18;10, and 19;9 in order of prevalence [99, 60, 200, 211], all resulting in the loss of *BRAF*'s regulatory domain. Subtle clinical differences between fusion variants have been noted but whether their underlying biology differs, and if additional roles of *KIAA1549* exist, remain unknown [57, 78, 116, 181].

**Fig. 3.**
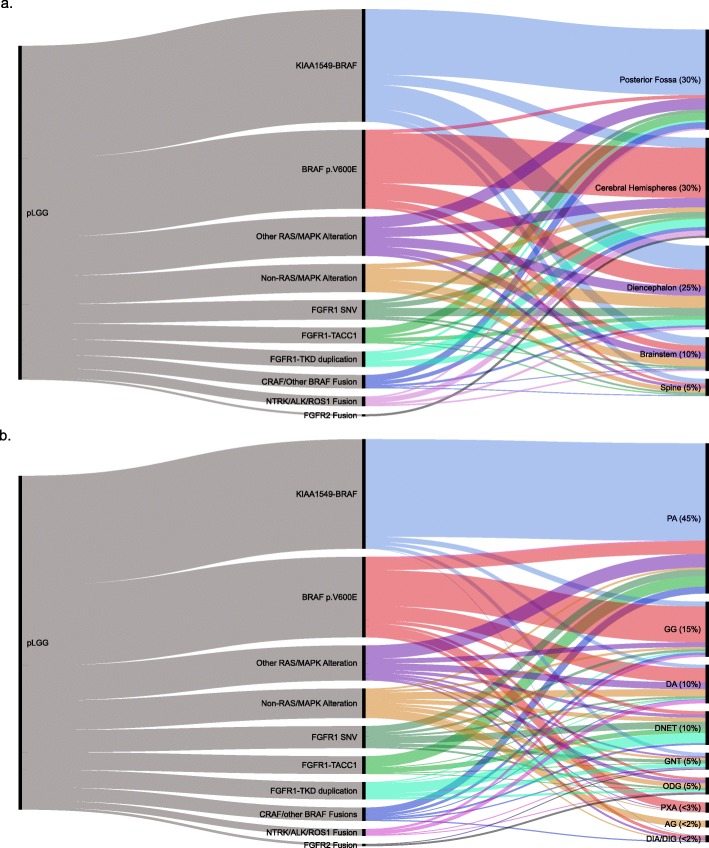
Distribution of molecular alterations as it pertains to **a**. tumor location and **b**. tumor histology. Plots were created using https://rawgraphs.io Accessed December, 2019

*KIAA1549-BRAF* is the most frequent molecular alteration in pLGG, and is significantly enriched in pilocytic astrocytoma and in tumors arising in the posterior fossa/cerebellum **(**Fig. 3a, b). Despite this enrichment, additional studies have confirmed *KIAA1549-BRAF* in a spectrum of histologies and CNS locations [60, 90, 97, 100, 116, 171, 200, 211, 229]. In total, *KIAA1549-BRAF* accounts for 30-40% of pLGG at the population level [186]. Due to its predilection for arising in highly circumscribed histologies (pilocytic astrocytoma) and in surgically amenable locations (cerebellum), tumors with a *KIAA1549-BRAF* fusion are often amendable to complete surgical resection and have excellent overall survival and rarely progress [11, 80, 84, 123]. However, when arising in deep seated regions of the brain where complete surgical resection is not possible, progression is more common [123]. The presence of *KIAA1549-BRAF* can aid in tumor diagnosis as it is not found in adult-type diffuse glioma and, with rare exceptions, confirms a pLGG diagnosis [78, 116, 178, 181]. Furthermore, it is helpful in identifying tumors susceptible to targeted therapeutics (discussed further below).

#### Other BRAF Fusions

In addition to *KIAA1549-BRAF*, *BRAF* rearrangements involving other fusion partners including *RNF130* [97], *SRGAP* [99], *FAM131B* [32], *CLCN6* [97], *GNAI1* [97], *MKRN1* [97], *GIT2* [81], and *FXR1* [229] among others have also been documented. As with *KIAA1549-BRAF*, these fusions result in the removal of *BRAF*'s N-regulatory domain and result in constitutive up-regulation of the RAS/MAPK pathway. As these fusions are extremely rare and often identified in isolated case studies, whether their impact on patient outcome differs from *KIAA1549-BRAF* remains unclear. However, in contrast to *KIAA1549-BRAF*, these non-canonical fusions are frequently observed in hemispheric and/or brainstem lesions and tend to arise in older children and adolescents. Further, despite also primarily arising in PA, they are also seen in an array of less common histologies [32, 60, 81, 97, 229]. Whether these unique clinical features are related to a different mechanism of tumorigenesis remains to be investigated.

#### BRAF p.V600E

Mutations in BRAF, primarily in which a valine is replaced with a glutamic acid at position 600 (p.V600E), act as a phosphomimetic within the RAS/MAPK pathway, rendering it constitutively active [64, 222]. In pLGG, the prevalence of the BRAF p.V600E mutation varies notably depending on the histology and location of the tumor **(**Fig. 3a, b). Pleomorphic xanthoastrocytoma (40-80%) [46, 68, 85, 191], diffuse astrocytoma (30-40%) [190, 191] and ganglioglioma (25-45%) [85, 123, 164, 191] frequently harbor BRAF p.V600E, while in pilocytic astrocytoma (5-10%) [85, 123, 191] or glioneuronal tumors (5%) [191, 47, 49], BRAF p.V600E is less common. Supratentorial lesions are also more likely to harbor BRAF p.V600E as compared to cerebellar lesions, while the inverse is true for *KIAA1549-BRAF* (Fig. 3a) [34, 46, 49, 171]. Importantly, despite these enrichments, BRAF p.V600E is neither histologically nor spatially restricted [47, 123, 171]. In addition to p.V600E, rare cases of BRAF p.V600D and BRAF p.V504_R506dup have been described in desmoplastic infantile astrocytomas/gliomas and pilocytic astrocytoma, respectively [27, 72, 107].

As a group, pLGGs with BRAF p.V600E have worse OS and PFS compared to other pLGG [28, 35, 50, 123, 158]. Further, BRAF p.V600E pLGG, especially in the context of co-occurring *CDKN2A* deletions (discussed further below), are significantly more likely to transform into HGG; an event which may occur 10-20 years after the initial diagnosis [142]. While not exclusive to this entity, transformation has been most commonly describe for pleomorphic xanthoastrocytoma which has been suggested to be within the same family as epithelioid GBM [4, 63, 210]. These "pleomorphic xanthoastrocytoma-like" GBM carry a better prognosis compared to other GBM, but are still significantly worse when compared to pLGG [117]. The increased likelihood of malignant behavior in pleomorphic xanthoastrocytoma and in BRAF p.V600E mutated tumors with *CDKN2A* deletion has led to a debate regarding the prognostic significance of BRAF p.V600E alone [101]. Future studies utilizing extensive cohorts with long-term follow-up will be required to address these questions conclusively.

#### FGFR1

FGFR1 is a receptor tyrosine kinase (RTK) that plays a key role in signal transduction via activation of its intramembranous tyrosine kinase domain (TKD) [69, 216]. While *FGFR1* mutations and/or fusions are only present in 3% of adult GBM [176, 202], it is the second most commonly altered gene in pLGG. *FGFR1* alterations in pLGG arise via three mechanisms: FGFR1 mutations, *FGFR1-TACC1* fusions and *FGFR1*-TKD duplications [97, 171, 229]. *FGFR1* mutations primarily consist of p.N546K and p.K656E and occur in 5-10% of patients [97, 171, 229]. As with *BRAF* alterations, these are histologically and spatially enriched, most frequently arising in dysembryoplastic neuroepithelial tumors, other glioneuronal tumors, and in midline brain structures (Fig. 3a-b). In these tumor subtypes, *FGFR1* mutations occur in up to 20% of patients, and in rare cases may be germline events [97, 136, 179, 191]. However, *FGFR1* mutations have also been reported in pilocytic astrocytoma, oligodendroglioma, and other histologies, and therefore are not histologically restricted [11, 67, 97, 171, 187, 229]. *FGFR1* TKD-duplication and *FGFR1-TACC1* fusions have each been described in 2-3% of tumors, [97, 171, 187, 229]. As with *FGFR1* mutations, *FGFR1* TKD-duplication is more common in dysembryoplastic neuroepithelial tumors and other glioneuronal tumors, while *FGFR1-TACC1* is more common in pilocytic astrocytoma. However, neither of these alterations are histologically restricted, also appearing in oligodendroglioma and diffuse astrocytoma, for example (Fig. 3a-b) [97, 171, 187, 229]. All of these alterations result in FGFR1 autophosphorylation [97, 229], leading to up-regulation of the RAS/MAPK pathway. In contrast to *BRAF* alterations, the upstream location of *FGFR1* (and the other receptor tyrosine kinase alterations described below) can result in up-regulation of the PI3K/AKT/mTOR pathway as well as depicted in Fig. 2a.

Despite being the second most common alteration in pLGG, the clinical manifestations of *FGFR1* alterations are still not well described. Becker *et al*., in their description of *FGFR1* mutations in pilocytic astrocytoma, noted that mutated tumors had a worse prognosis than their wild-type counterparts [11]. Whether this worsened outcome is due to the alteration itself or the propensity for *FGFR1* mutated tumors to arise in the midline is unknown. Importantly, *FGFR1* mutations often contain additional alterations, most frequently a second event in *FGFR1* resulting in an *FGFR1* “dual hit” [97, 229]. In addition, co-occurring alterations in *BRAF*, *KRAS*, *NF1*, *PTPN11* and *H3F3A* have also been reported [97, 171, 187, 229]. Except for *H3F3A*, whether these additional alterations impact patient prognosis is yet to be established. However, the propensity for *FGFR1* mutations (but not TKD duplication or *FGFR1-TACC1*) to co-occur with additional alterations is interesting and may provide insight into the underlying pathogenesis of these tumors.

#### CRAF Fusions

Fusions involving *CRAF* (*RAF1*), a human homolog of the v-raf gene implicated in cell proliferation and survival, are infrequently identified in pLGG, most commonly in pilocytic astrocytoma. These include *QKI-RAF1* [131, 229], *FYCO-RAF1* [229], *TRIM33-RAF1* [43], *SRGAP3-RAF1* [98, 99], and *ATG7-RAF1* [95, 96] among others. As with non-canonical *BRAF* fusions, *CRAF* fusions have been shown to up-regulate the RAS/MAPK pathway [93, 98, 99]. Due to the rarity of *CRAF* fusions, their clinical implications are unclear.

#### NTRK Fusions

The neurotrophic tyrosine receptor kinase (*NTRK*) family of genes play key roles in CNS development [75, 213, 219] and has long been implicated in a variety of cancers [192, 213]. *NTRK* fusions have been identified in various histological subtypes of pLGG, albeit at very low frequencies. These alterations include *SLMAP-NTRK2, TPM3-NTRK1, ETV6-NTRK3* and *RBPMS-NTRK3* [97, 171, 215, 229]. All these fusions are predicted to drive tumorigenesis via aberrant dimerization of the *NTRK* kinase domain, resulting in constitutive downstream activation that, at least in part, impacts both the RAS/MAPK and PI3K/AKT/mTOR pathways [104, 108, 148]. These results have led to several clinical trials using targeted agents against *NTRK* (discussed below).

#### KRAS Mutations

A small subset of non-*BRAF* mutated pLGG harbor mutations in *KRAS*, an upstream molecule in the RAS/MAPK pathway (Fig. 2). Reports on the frequency of *KRAS* mutations in pLGG range from 1-5% and primarily arise in pilocytic astrocytoma [94, 95, 97, 164, 229]. Most frequently, these are p.G12D or p.Q61H/K, although one report noted both novel and dual *KRAS* mutations within 2 patients [97]. Importantly, *KRAS* mutations are also seen in high grade gliomas and therefore cannot be used as a diagnostic marker for pLGG. Given the success of inhibiting downstream effectors of *KRAS* mutations in other cancer types [74], identifying these mutations in pLGG may offer access to targeted treatment approaches.

#### PTPN11 Mutations

*PTPN11* (or *SHP-2*) is a tyrosine phosphatase adaptor protein within the RAS/MAPK pathway known to cause Noonan syndrome [182]. With regards to pLGG, specifically pilocytic astrocytoma, *PTPN11* alterations have been reported in approximately 2% of cases [95, 97]. Interestingly, in these studies 82% of *PTPN11*-mutant cases also harbored alterations in *FGFR1*, suggesting that the two are biologically linked. In the original report defining the mutation, the authors noted that *PTPN11* over-expression alone did not significantly activate the RAS/MAPK pathway, but did when in the presence of *FGFR1* mutations [97]. The authors suggested that *PTPN11* alone was insufficient to promote transformation, but instead played a modifying role in *FGFR1*-mutant pLGG. Future work in GBM proposed that *PTPN11* is essential for maintaining a glioma stem cell population during transformation [180] and for activating PI3K/AKT/mTOR signalling [129]. This suggests that mTOR inhibitors may be more effective than RAS/MAPK inhibitors in pLGG harboring these alterations.

#### ALK Fusions

The anaplastic lymphoma kinase (*ALK*) gene is thought to play a key role in the development and function of the nervous system and chromosomal alterations and gain of function mutations in it have been reported in a plethora of pediatric cancers [29, 30, 106, 137, 220]. These alterations are most commonly fusion events that result in ectopic expression of the ALK fusion protein [6]. This results in up-regulation of the RAS/MAPK and PI3K/AKT/mTOR pathways [73, 143]. Despite the frequency of *ALK* alterations in pediatric cancer, reports of its presence in glioma are rare and often exist in isolated case reports [1, 147, 152]. The most frequently reported alterations are *CCDC88A-ALK* and *PPP1CB-ALK*, both resultant fusions from a larger chromothripsis event [1, 73, 147, 152]. Recently, *ALK* alterations were shown to form a unique clinical subgroup of infantile glioma that require would likely benefit from a refined treatment approach [73].

#### ROS1 Fusions

*ROS1* is an orphan tyrosine receptor with no known ligand nor definitive function despite speculation for a role in cell proliferation and differentiation. In pLGG, *GOPC-ROS1* is the result of an intrachromosomal deletion that results in a constitutively active kinase fusion product sufficient to promote neoplastic transformation both *in vitro* and *in vivo* [26, 40]. Although *GOPC-ROS1* represents the most common *ROS1* alteration in gliomas, *CEP85L–ROS1*, *ZCCHC8-ROS1*, and *KLC1-ROS1* have also been reported [33, 40, 146]. The use of targeted agents against *ROS1* in lung cancers has shown dramatic clinical efficacy [54, 198], which has resulted in interest regarding their use in glioma.

#### MAP2K1 Alterations

Alterations including p.Q56P and small in-frame deletions in *MAP2K1* were frequent in a small cohort of multinodular and vacuolating neuronal tumors (MVNT) [163]. Within pLGG, this alteration appears to be enriched for this histological subtype, as follow-up work looking into the molecular landscape of ganglioglioma did not identify any further *MAP2K1* alterations [164]. However, *MAP2K1* is altered in other non-pLGG tumors including lung and colorectal cancers and thus, as with *KRAS*, is not specific to these entities. These alterations in other malignancies have shown up-regulation of the RAS/MAPK pathway and may have a similar mechanism in MVNT [18, 23, 156].

#### Other Rare RAS/MAPK Alterations

Recurrent alterations involving *FGFR2/3* (rather than the more frequent *FGFR1*) have been identified in a recently defined tumor type, PLNTY, which carries a good prognosis [88]. These occur exclusively as fusion events, most commonly as *FGFR2-KIAA1598* and *FGFR2-CTNNA3* but also rarely as *FGFR3-TACC3*. In contrast to *FGFR1-TACC1*, *FGFR3-TACC3* is extremely rare in pLGG, but arises in ~3% of IDH1/2 wild-type adult GBM. Therefore, paying close attention to the histologic features is important for tumors harboring this fusion [45, 152].

PDGFRα mutations have been reported in low grade glioneuronal tumors of the septum pellucidum [204], despite more typically being associated with HGG in the context of other mutations [109, 193, 207, 226, 227]. The clinical implications of these rare alterations are not yet fully understood.

### Non-RAS/MAPK Related Alterations in pLGG

The degree of molecular data converging on the RAS/MAPK pathway has justifiably led to speculation that pLGG is a “one-pathway” disease [34, 96, 149, 229]. However, despite this, several alterations with seemingly no direct impact on RAS/MAPK signalling have also been described. It may be that these aberration do, in fact, impact this pathway via mechanisms not yet discovered. We discuss these non-RAS/MAPK alterations below.

#### MYB alterations

Myb proto-oncogene protein (*c-MYB*) is a member of the myeloblastosis family of transcription factors named after the avian myeloblastosis virus gene (v-Myb) which causes myeloid leukemia in chickens. It plays an important role in the control of proliferation and differentiation of hematopoietic and other progenitor cells and has well described proto-oncogenic functions in both human leukemia and solid tumors where it is thought that super-enhancers to *c-MYB,* as a consequence of chromosomal translocation, cause overexpression of *c-MYB* [160, 230]. *MYB*’s involvement in pLGG was first described in 2010 by Tatevossian *et al.* who identified *MYB* amplification in 2 of 14 diffuse astrocytomas and a focal deletion of the terminal region of *MYB* in 1 of 2 angiocentric gliomas [212]. The authors concluded that 60% of diffuse astrocytomas displayed MYB up-regulation at the protein level, but were unable to identify a unifying genetic event responsible for the observation. This finding was later confirmed, when 22% (8/36) of diffuse cerebral gliomas, including diffuse astrocytoma and angiocentric glioma, were shown to have a *MYB* 3’ truncating fusion or, less commonly, amplification resulting in elevated expression at the protein level [229]. More recently, Bandopadhayay *et. al.* published that 10% (16/172) of their pLGG cohort contained *MYB* alterations, most commonly as *MYB-QKI* fusions, including 19/19 (discovery and validation cohorts) angiocentric gliomas [8]. This fusion was shown to likely function via a tripartite mechanism of MYB protein activation, *MYB* overexpression and the loss-of-function of *QKI* [8]. Work investigating the genetics of uncommon low-grade neuroepithelial tumors showed that 87% and 41% of angiocentric glioma and diffuse astrocytoma, respectively, harbored *MYB* alterations [171]. *MYB-ESR1*, *MYB-PCDHGA1*, *MYB-LOC105378099, MYB-MMP16*, *MYB-LOC154902*, and *MYB-MAML2* in addition to *MYB-QKI* have also been identified [31, 171, 229]. Importantly, *MYB* alterations are histologically restricted to angiocentric and diffuse gliomas.

#### MYBL1 alterations

*MYBL1* (MYB Proto-Oncogene Like 1) is a closely related family member of *MYB*, and is thought to likewise act as a transcriptional regulator critical for proliferation and differentiation. Although commonly grouped together due to their overlapping biological function, much less is known about *MYBL1* compared to *MYB*-driven tumors. Originally described in Ramkissoon *et. al.* in 28% (5/18) of diffuse astrocytomas, these *MYBL1*-driven tumors showed a partial duplication with truncation of its C-terminal regulatory [174]. The common breakpoint immediately preceding the C-terminal regulatory domain in these cases suggest the potential formation of a functional, truncated gene product. However, the concise downstream functional consequence of this event remains to be fully elucidated [174]. More recent reports of *MYBL1* alterations in pLGG suggest *MYBL1* alterations may be even rarer, being found in 2/17 (12%) [171], 7/50 (14%) [8], and 1/17 (6%) [229] diffuse astrocytomas. No other histological diagnoses have been reported to harbor *MYBL1* alterations.

*MYB* and *MYBL1* alterations were originally described in diffuse gliomas of childhood. They are more likely to arise in young children (median age 5 years) and are significantly enriched for the cerebral hemispheres, although infrequently they occurred in the diencephalon or brainstem [31, 25, 38]. A recent single-centre pediatric study showed a 10-year OS and PFS of 90% and 95%, respectively, suggesting that these lesions are indolent [31]. These alterations have also been described in the adult age group where they represented ~50% of so-called isomorphic diffuse glioma (a subtype of *IDH1* wild-type, BRAF p.V600E negative diffuse astrocytoma) in both children and adults [223]. These tumors, despite their diffuse astrocytoma morphological features, had a good prognosis. When clustered on t-SNE via methylation analysis, both *MYB* and *MYBL1* tumors cluster together, and the authors conclude that they reflect a single tumor entity [31]. However, this hypothesis merits further investigation as more of these rare cases, in particular those harboring *MYBL1* alterations, are reported.

#### IDH1 Mutations

Mutations in *IDH1* are present in ~70% of grade II, grade III, and secondary GBM in adults, most frequently at position p.R132 [7, 15, 77, 228]. Despite their frequency in adults, *IDH1* mutations in pediatric glioma are rare, with reports ranging from 0-17% of cases [7, 41, 77, 169, 228]. As with adult tumors, the *IDH1* mutation is usually in the context of either 1p/19q co-deletion or is associated with *TP53* and *ATRX* mutations and as such, likely represent the lower end of the age spectrum of adult-type IDH-mutant glioma [103, 130]. There is a significant correlation between IDH1 alterations and patient age. In one report, *IDH1* mutations were identified in 5% of pediatric gliomas which collectively had a median age of 16 [41]. Likewise, a report from the Children’s Oncology Group noted a 16% incidence of *IDH1* mutations, all of which occurred in patients over the age of 14 [169]. In adults, *IDH1* mutations are associated with a better prognosis and response to chemotherapy as compared to *IDH1/2* wild-type glioma [76, 86, 140, 151, 188]. While the clinical impact of *IDH1* mutations in children is far less understood, it is likely that they will not behave in the same indolent way as most other pLGG over the long term. It is plausible that these tumors are in fact adult malignancies that have been identified early. As such, these tumors should be more closely followed than true pLGG [92].

#### H3F3A Mutations

Mutations in histone variant *H3F3A* (H3.3) were first described in pediatric high grade glioma, specifically diffuse intrinsic pontine glioma (DIPG), where they are present in approximately 65% of tumors [109, 134, 193, 207, 226, 227]. H3.3 p.K27M is exclusively observed in tumors arising in the midline, including the pons, diencephalon/thalamus and spinal cord [66, 109, 134, 193, 207, 226, 227]. Although more frequent in HGG, H3.3 p.K27M has been reported in pLGG including pilocytic astrocytoma [82, 153], ganglioglioma [102, 112, 158] and diffuse astrocytoma [187, 205]. In one series of pediatric thalamic glioma, H3.3 p.K27M was noted in 12% of low grade cases [187]. Interestingly, H3.3 p.K27M has been shown to co-occur with additional hotspot mutations, including BRAF p.V600E, FGFR1 p.N546K or p.K656E, and *NF1* mutations [102, 112, 158, 187]. Patients with H3.3 p.K27M pLGG have the potential to live longer than patients with H3.3 p.K27M glioma with high grade histologic features. Indeed, there are reports of survival of up to 10 years post-surgery in rare cases [82, 102, 112, 153 158], although most patients succumb to their disease within 1-3 years. In this regard, despite their comparatively longer survival, these tumors tend to mimic the clinical impact of H3.3 p.K27M in HGG in that they invariably progress and cause death, starkly contrasting the excellent survival of non-H3.3 p.K27M mutant pLGG as described above.

### Secondary Alterations in pLGG

#### *CDKN2A* Deletion

Homozygous and hemizygous losses involving 9p21 are frequent in adult infiltrating glioma and GBM [13, 150, 163]. One of the consequences of this deletion is the loss of the tumor suppressor *CDKN2A*, which endogenously functions as a G1 cell-cycle regulator [125, 184]. Homozygous deletion of *CDKN2A* is also observed in pLGG, albeit at a lower frequency than in adult glioma [10, 161, 162, 165, 170]. Reports suggest that *CDKN2A* loss ranges in frequency from 6-20% in pLGG, with significant enrichment in pleomorphic xanthoastrocytoma [17, 51, 84, 190]. Likewise, *CDKN2A* deletion frequently co-occurs with BRAF p.V600E, suggesting that it likely acts as a second molecular hit, promoting escape from cell cycle regulation [17, 84, 85, 91, 173, 190]. Tumors harboring both BRAF p.V600E and *CDKN2A* deletion comprise a distinct clinical subtype of pLGG prone to transformation into secondary HGG [142]. This is in line with reports showing that co-occurrence of *CDKN2A* deletion with BRAF p.V600E is associated with escape from oncogene-induced senescence [91, 173] and with having a worse OS and PFS [85]. Interestingly, several reports have also shown that pediatric grade I gliomas harboring *CDKN2A* loss, despite their rarity, have a more aggressive clinical course consistent with that of a higher histological grade [173, 190] and co-occurrence of *CDKN2A* deletion with *BRAF* fusions has been described in anaplastic astrocytoma with piloid features [178]. As such, pLGG with *CDKN2A* deletions, especially in the context of BRAF p.V600E or with possible high grade histologic features, should be considered as high risk tumors that warrant close clinical follow-up.

## Molecular Tests and Platforms for profiling pLGG

Currently, a wide array of clinically-certified laboratory methods are used to molecularly profile pLGG. However, no "gold standard" exists for testing the array of potential molecular events and various strategies may be used depending on tissue quality/quantity and budget. As detailed above, one should strive to have tools which can identify SNVs and gene fusions. Simple and robust tests which can be used to detect common alterations such as *BRAF* fusions and BRAF p.V600E allow molecular characterization of almost two thirds of pLGG.

Below, we discuss some of the common testing strategies used to molecularly profile pLGG and include their tissue requirements, cost, turn-around time, and target-specific applicability **(**Table 2**)**.

**Table 2:** Common testing techniques utilized to molecularly characterize pLGG including their associated cost, input requirements, limitations and time requirements

Technique	Time (hours)	Cost (per sample)	Input	Utility	Clinical Limitations
Immunohistochemistry	+	+	1 FFPE slide	1 target/slide	• Subject to antibody availability
Fluorescent in situ hybridization	++	++	1 FFPR slide	1 target/slide	• Subject to probe design/ availability
Droplet digital PCR	+	+	10-50ng DNA/target	1 target per reaction	• Requires access to expensive equipment
NanoString nCounter	++	++	200-500ng RNA	Up to 800 targets per reaction	• Requires RNA <10 years old • Requires access to expensive equipment
SNP Array	+++	+++	100-200ng of DNA	Dependent on probe frequency	• Limited to copy number alterations • Subject to batch effect
Next Generation Sequencing Panels	+++	+++	20-100ng DNA/RNA	Design dependent	• Requires RNA <10 years old • Requires access to expensive equipment • Requires significant downstream analysis
Methylation Array	+++	+++	20-50ng of bisulphite converted DNA	Methylation-based diagnosis [20]	• Requires access to expensive equipment • Subject to batch effect

### Immunohistochemistry

Immunohistochemistry (IHC) is a simple and robust test which can identify specific alterations in most laboratories. IHC is capable of detecting protein specific expression indicative of the tumor's underlying mutational status in a timely, cost-effective manner while requiring very little tissue in the process. With respect to pLGG, IHC has been faithfully utilized in the detection of BRAF p.V600E [21], H3.3 p.K27M [221] and IDH1 p.R132H [22] and can be used on formalin-fixed-paraffin-embedded (FFPE) tissue. However, this approach is limited to those alterations with available antibodies.

### Fluorescent in situ Hybridization

Fluorescent in situ hybridization (FISH) allows for visualization of both gene fusions and copy number events at a single cell resolution. In pLGG, FISH has been used in the identification of *BRAF* [214], *FGFR1* [179], *ALK/ROS1/NTRK1/2/3* fusions [73], and *MYB/MYBL1* alterations [174, 212]. It can also be used for identifying co-occurring *CDKN2A* deletions. FISH is widely available and can be used on FFPE material but is relatively labor-intensive, and can only test for a single alteration at a time.

### Droplet Digital PCR

Point mutations can be also detected using polymerase chain reaction (PCR) techniques. If available, the advantage of droplet-digital PCR (ddPCR) is its ability to faithfully detect mutations at very low variant allele frequencies related to low quality or highly diffuse input material. In addition, ddPCR's ability to do high throughput testing makes the test affordable when run at capacity. In this process, single fragments of DNA are partitioned into oil-based droplets and amplified using standard Taq-Man probes designed against the desired target [172]. As each individual droplet is devoid of competition, each DNA fragment is amplified, allowing for unparalleled sensitivity. ddPCR can identify not only point mutations such as BRAF p.V600E [123], H3.3 p.K27M [187], IDH1 p.R132H [187], and FGFR1 p.N546K and p.K656E [58] but also *CDKN2A* deletions [123], *KIAA1549-BRAF* [5], and *FGFR1* TKD-duplication [58] based on copy number comparisons. This technique is very robust on degraded DNA, including from FFPE material, and requires minimal technical hands-on time. However, it is difficult to multiplex and requires access to expensive equipment.

### NanoString nCounter

The NanoString nCounter system is a hydridization based platform capable of detecting fusion transcripts in a multiplexed fashion [186]. NanoString panels can be used to screen for the common fusions such as those involving *BRAF* (including both the canonical *KIAA1549-BRAF* and the non-canonical fusions described above) and *FGFR1-TACC1* [186] as well as for rarer fusions including those involving *ALK*, *ROS1*, *NTRK*, and *MET* [73]. This technology is robust on FFPE material, requires minimal technical hands-on time and bioinformatic analysis is relatively simple. However, input requirements are relatively high (200-500ng of RNA), the fusion partner and exact breakpoint must be known and it requires access to expensive equipment.

### SNP Array

In cases where no specific alterations can be found using the gene specific tools, or when copy number alterations have a role in tumor management, genome-wide SNP arrays can be used. SNP arrays are a probe-based molecular profiling technique optimized for the detection of copy number variants. Their use in pLGG molecular profiling includes the identification of *BRAF* and *FGFR* fusions, *MYB* and *MYBL1* alterations and *CDKN2A* deletions. SNP arrays are robust with FFPE material, but require expensive reagents, long technical hands-on time and batching of samples as well as a moderate amount of input material (100-200ng of DNA) and access to expensive equipment.

### Next generation sequencing panels

In recent years, the use of next-generation sequencing (NGS) platforms for the molecular characterization of solid tumors has gained significant popularity [113, 175, 209]. These platforms range from approximately 300-500 gene targets (or more) and often include most of those altered in pLGG. Sequencing based approaches have the benefit of simultaneous detection of most clinically relevant alterations in a single test from which diagnostic, prognostic and therapy decisions can be made. However, tissue quality requirements, which are generally higher as compared to the other technologies, technical hands-on time and downstream analysis are more complicated and time-consuming leading to longer turn-around-times and cost. Access to expensive equipment is also required, all of which limit the use of these approaches globally. Nevertheless, in the cases where the tools above cannot identify the pLGG molecular driver, NGS approaches are highly advantageous.

### Methylation Profiling

DNA methylation profiling is another tool which can aid in the diagnosis of tumors arising in the CNS [20]. This method is particularly useful in aiding the diagnosis of difficult tumor entities and is robust on FFPE material. In addition, current arrays can detect copy number alterations, albeit at a lower resolution than SNP arrays. However, the utility of the methylation classifier may be less robust in pLGG, possibly due to frequent inclusion of normal tissue in these tumors [20]. Furthermore, methylation profiling remains expensive, is subject to batch effects, and must be run in sets of 8. Further, the utility of methylation profiling as it pertains to tumor diagnosis requires further investigation.

## Molecular pLGG diagnostic algorithm

**Fig. 4.**
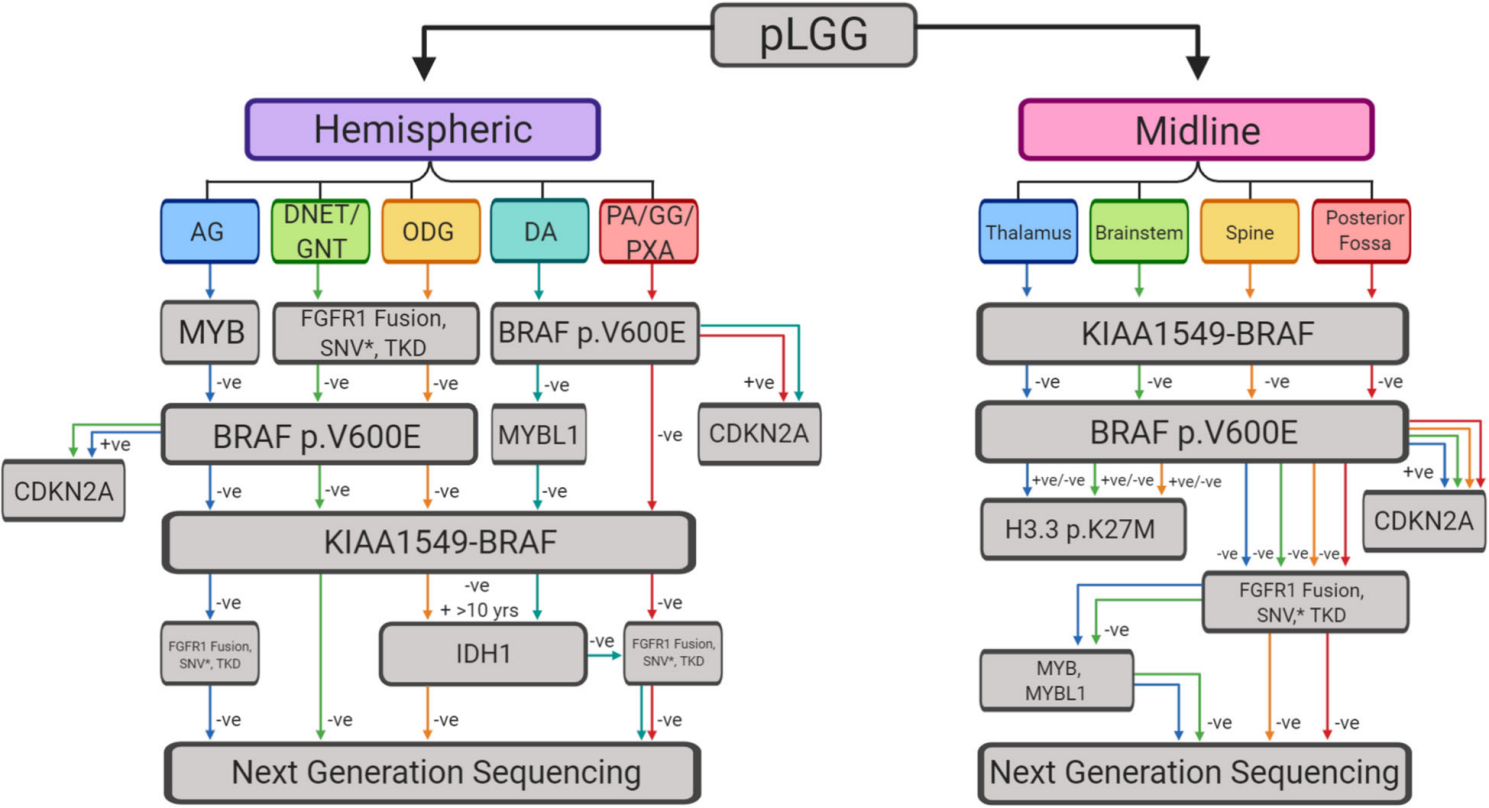
Molecular testing decision tree for pediatric low-grade glioma. *The frequency with which tumors harbor an *FGFR1* mutation and additional mutations justifies continued testing regardless of status. AG: angiocentric glioma, DNET: dysembryoplastic neuroepithelial tumour, GNT: glioneuronal tumor, ODG: oligodendroglioma, PA: pilocytic astrocytoma, GG: ganglioglioma, PXA: pleomorphic xanthoastrocytoma, DA: diffuse astrocytoma

Given the array of molecular alterations and their overlap amongst different tumor histologies, devising a simple testing recommendation for pLGG can be difficult. Ultimately, as proposed by Miklja *et. al.* [141], there are two primary approaches to the problem (i) sequential testing of specific alterations in a tier-based approach or (ii) upfront NGS panels optimized for pLGG. The latter may be used in centers with access to this technology and for whom cost is not an issue. For other centers the use of a sequential testing strategy is largely supported by the fact that the vast majority of pLGG harbor a single molecular driver within a subset of recurrently altered genes. These events primarily occur as either gene fusions or mutations, but almost never both. Rarely, exceptions arise where multiple mutations, either within the same gene or in other traditional pLGG targets, arise. These are almost exclusively observed with mutations and not gene fusions. The crux of this strategy lies in its ability to accurately identify a molecular driver prior to the number of tests conducted exceeding the cost and turn-around-time of an NGS based approach. A possible testing strategy highlighting the most probable molecular alterations present based on the tumor's clinical features is included in Fig. 4.

## Targeted Molecular Therapies for pLGG

### BRAF Inhibitors

First generation *BRAF* inhibitors including dabrafenib and vemurafenib have shown excellent results in melanoma patients harboring BRAF p.V600E and are now being investigated for their utility in pLGG [79, 206]. A series of case reports utilizing these agents in a single agent approach showed excellent results with most reporting a complete response [2, 24, 42, 121, 185, 203]. These findings were recently confirmed in a larger cohort of BRAF p.V600E tumors, in which either of these BRAF inhibitors induced significant cytoreduction and prolonged survival in patients [123]. These results led to a multi-institute phase I clinical trial, where initial findings using dabrafenib reported an impressive overall response rate of 41% [110] A follow-up trial optimizing the dosing safety and tolerability is currently underway (NCT01677741). Despite their efficacy in BRAF p.V600E tumors, first generation *BRAF* inhibitors result in paradoxical activation of RAS/MAPK signalling when used in *KIAA1549-BRAF* or *BRAF* wild-type tumors [201]. This was the case in a trial of sorafenib, which caused accelerated tumor growth and resulted in the early termination of the trial [105]. To rectify this issue, second generation "paradox-breaker" agents were designed to inhibit *BRAF* without causing paradoxical RAS/MAPK activation [217]. Of note, *CRAF* fused pLGG were unresponsive to both first and second generation *BRAF* inhibitors [93]. This was attributed to the robust protein-protein interactions mediated by the *CRAF* fusion partners [93]. This highlights the necessity of careful molecular characterization of pLGG prior to making treatment decisions, and emphasizes the risk of conducting trials without proper molecular characterization.

### MEK Inhibitors

For pLGG that are not suitable for type I BRAF inhibitors (NF1-pLGG, *KIAA1549-BRAF* fused, etc.), MEK inhibition has emerged as a promising therapeutic strategy. Currently, four MEK inhibitors including selumetinib [9, 56)], trametinib (NCT03363217), cobimetinib (NCT02639546), and binimetinib (NCT02285439) are at various stages of clinical testing. For selumetinib, both phase I and II trials have been completed [9, 56]. The phase I study focussing on NF1-associated and sporadic refractory or progressive pLGG showed that 32/38 patients exhibited either stability or reductions in tumor size [9]. Similar results were seen in a Phase II study, where use of selumetinib in recurrent pLGG boasted impressive results, with 40% of NF1 patients achieving partial response and only 1 patient progressing while on treatment [56]. Given these positive results, efforts to evaluate the use of selumetinib upfront in newly diagnosed patients both as a single agent or in combinations are under way. The trial of trametinib involving 6 patients resulted in 2 partial and 3 minor responses, while 1 patient had progressive disease [115].

### FGFR1 Inhibitors

Due to its functional importance and frequent implications in cancer, multiple small molecular inhibitors of *FGFR* have been developed, some of which are in clinical trials for an array of malignancies. These include AZD4547 (NCT02824133) for treatment of malignant glioma harboring FGFR-TACC fusions [65] and several others previously reviewed [36]. Results from these trials will inevitably influence the applicability of these agents in pediatric glioma.

### ALK/ROS1/NTRK Inhibitors

Alterations in *ALK*, *ROS1* and *NTRK* are relatively rare in pLGG. Conveniently, alterations in these genes are common in adult malignancies including lung and colorectal cancer and as such, targeted agents with federal approval have already been developed and tested. These include Crizotinib (NCT00939770) [144], ceritinib (NCT02336451) [111], and cabozantinib (NCT00704288) [224], as well as many investigational agents, such as brigatinib (ALK/ROS1) [39], entrectinib (ROS1/TRK) [128], and larotrectinib (TRK) [83, 194], the latter of which was recently approved in the treatment of TRK-altered cancers (NCT02122913) [83]. In pediatric glioma specifically, both entrectinib and larotrectinib have shown potent anti-tumor effects (NCT02637687, NCT02576431) [52, 53, 120]. These results have led to a current phase I/Ib study being conducted in pediatrics to evaluate Entrectinib in primary CNS tumors (NCT02650401).

## The Future of pLGG Classification

The importance of molecular testing in tumor diagnostics is increasingly recognised and became formalised for brain tumors in the most recent WHO classification [132]. As we gain a better understanding of the molecular underpinnings of pLGG, it is becoming evident that, while certain histologies may be enriched for particular molecular events (and vice versa), they are not exclusively associated with a particular event. Furthermore, while classic morphologies exist for the entities encompassed within the umbrella of pLGG, there remain cases with overlapping features between histologic categories, as was discussed above. Importantly, whether a particular molecular event carries the same prognostic significance across different pLGG entities is currently unclear. Given this, a layered diagnostic approach is recommended where both the histologic classification and molecular findings are reported in an integrated diagnosis [133]. Most importantly, pLGG need to be distinguished from their adult-type counterparts as both clinical management and long term outcome are drastically different.

**Fig. 5.**
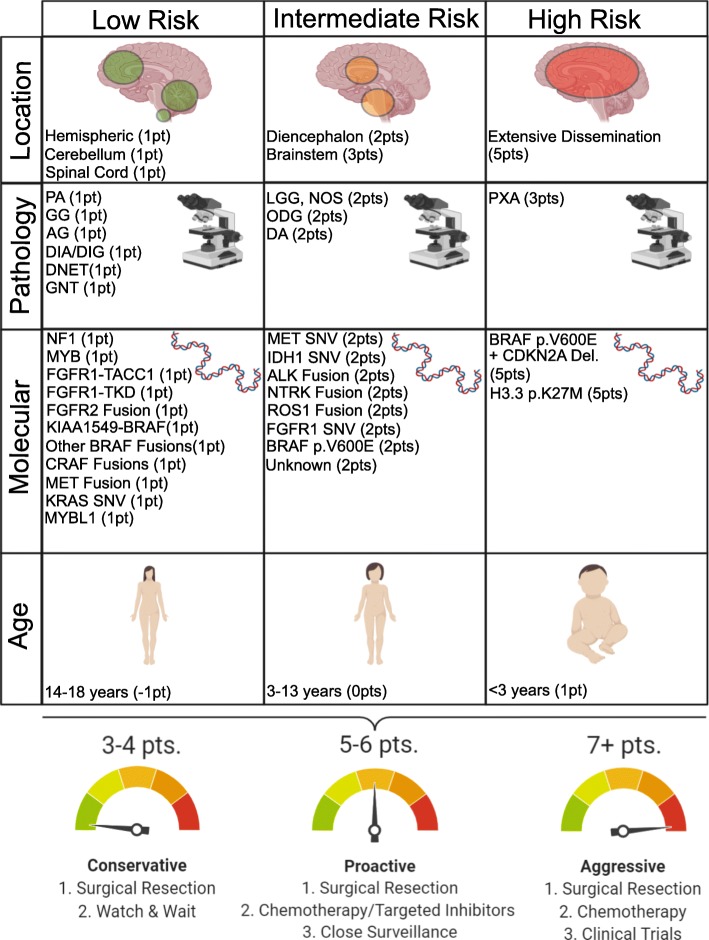
Risk association for clinical and molecular features of pediatric low-grade glioma. Associated points are to be totaled for tumor location, histology, age at diagnosis and molecular driver. Point totals denote a risk association accompanied with clinical suggestions for proper tumor management. AG: angiocentric glioma, DNET: dysembryoplastic neuroepithelial tumour, GNT: glioneuronal tumor, ODG: oligodendroglioma, PA: pilocytic astrocytoma, GG: ganglioglioma, PXA: pleomorphic xanthoastrocytoma, DA: diffuse astrocytoma, DIA/DIG: Desmoplastic infantile astrocytoma/ganglioglioma, LGG, NOS: low-grade glioma, not otherwise specified

A comprehensive risk based classification of pLGG lies in an integrated model, utilizing clinical, imaging and molecular information to concisely categorize tumors based on their potential clinical risk (Fig. 5) [95, 97, 123, 171, 229, unpublished data]. The scheme we propose here attempts to incorporate these factors into one tool. For example, a pLGG with typically benign histology, a *KIAA1549-BRAF* fusion, and arising in a child between 3-12 years would typically be viewed as low risk and a "watch and wait" strategy may be employed, followed by less aggressive therapies if the tumor were to progress. In contrast, a tumor in an unfavorable location or highly disseminated with high risk molecular features will require close clinical follow-up and a more aggressive therapeutic approach (Fig. 5). This schematic approach would also allow for amendments incorporating adjunct strategies such as the methylation classifier [20] or other novel molecular targets upon their discovery.

## Conclusion

The era of precision medicine for pLGG has arrived. Molecular stratification of pLGG resulting in significant clinical implications is currently available and has been seen in trials for specific inhibitors such as BRAF p.V600E- and MEK-inhibitors. The expected availability of FGFR-targeted agents, as well as other tyrosine kinase inhibitors for rare fusions, makes precision diagnostics key to the management of these patients. Indeed, the current National Cancer Institute–Children’s Oncology Group Pediatric MATCH trial (NCT03155620) aims to match actionable mutations to 9 investigational therapies, providing a glimpse into the future of pLGG treatment. In this context it is important to be aware of which methods are available to be used that are not reliant on expensive NGS-based technologies, and here we provide a testing pipeline to aid in testing decisions. Importantly, molecular stratification is only one factor influencing the behavior of pLGG. Other factors such as age, tumor location, and histopathology are required to inform a comprehensive approach to prognostication and treatment of pLGG. We therefore propose a pLGG risk classification schema that utilizes the breadth of clinical and molecular information available to best equip clinicians as we transition to this new era of pLGG classification and treatment.

## Data Availability

The source of all data and material are cited in the manuscript. Availability of these are governed by the originating authors and source.

## Abbreviations

CNS: Central nervous system
ddPCR: Droplet-digital polymerase chain reaction
DIPG: Diffuse intrinsic pontine glioma
FFPE: Formalin-fixed-paraffin-embedded
FISH: Fluorescent in situ hybridization
GBM: Glioblastoma
HGG: High-grade glioma
H&E: Hematoxylin and eosin stain
IHC: Immunohistochemistry
LGG: Low-grade glioma
MRI: Magnetic resonance imaging
MVNT: Multinodular and vacuolating neuronal tumors
NF1: Neurofibromatosis Type I
NGS: Next generation sequencing
NTRK: Neurotrophic tyrosine receptor kinase
OS: Overall survival
PCR: Polymerase chain reaction
PFS: Progression-free survival
pLGG: Pediatric-type low-grade glioma
PLNTY: Pleomorphic neuroepithelial tumor of the young
RAS/MAPK: RAS-mitogen-activated protein kinase
RTK: Receptor tyrosine kinase
SNV: Single nucleotide variant
TKD: Tyrosine kinase domain
WHO: World Health Organization
